# Recent insights into the effects of metabolism on breast cancer cell dormancy

**DOI:** 10.1038/s41416-022-01869-5

**Published:** 2022-06-17

**Authors:** Sara Bartlome, Catherine Cecilia Berry

**Affiliations:** Centre for the Cellular Microenvironment, Advanced Research Centre, College of Medical, Veterinary & Life Sciences, University of Glasgow, 11 Chapel Lane, G11 6EW Glasgow, UK

**Keywords:** Breast cancer, Cancer metabolism, Bone metastases, Cell-cycle exit

## Abstract

Breast cancer (BC) remains the most common cancer, as well as the leading cause of cancer mortality in women worldwide [1]. Approximately 30% of patients with early-stage BC experience metastasis or a recurrent form of the disease [2]. The phenomenon of BC dormancy, where metastasised cancer cells remain in a quiescent phase at their disseminated location and for unknown reasons can become actively proliferative again, further adds to BC’s clinical burden with treatment at this secondary stage typically proving futile. An emerging avenue of research focuses on the metabolic properties of dormant BC cells (BCCs) and potential metabolic changes causing BCCs to enter/exit their quiescent state. Here we explore several studies that have uncovered changes in carbon metabolism underlying a dormant state, with conflicting studies uncovering shifts towards both glycolysis and/or oxidative phosphorylation. This review highlights that the metabolic states/shifts of dormant BCCs seem to be dependent on different BC subtypes and receptor status; however, more work needs to be done to fully map these differences. Building on the research that this review outlines could provide new personalised therapeutic possibilities for BC patients.

## Background

Despite advances in early diagnostics, anti-cancer therapeutics and improved patient outcomes, breast cancer (BC) remains a clinical problem [3]. The charity Breast Cancer Now estimates that the number of women dying from BC each year in the UK is set to rise in 2022, with BC currently proving fatal for nearly 1000 women in the UK each month [4]. BC is a highly multifaceted disease, with patients presenting with a variety of different physiological and morphological phenotypes. Thereby, clinical emphasis is directed at the molecular classification of BC subtypes, namely luminal A, luminal B, human epidermal growth factor receptor 2 (HER2) enriched and basal like, each exhibiting their own prognostic implications (see Table 1) [5].

**Table 1:** BC subtypes, their receptor phenotypes and prognostic implications.

BC subtype	Receptor status	Frequency^a^	5-year overall relative survival rate^a^	5-year distant metastatic relative survival rate^a^	Common sites of BC metastasis	Frequency of cancer subtype at site of metastasis^b^	Notes
Luminal A	ER+ PR+ HER2−	68%	94.3%	30.6%	Bone	58.5%	Subtype with the best prognostic chance of survival Subtype that occurs most frequently
					Brain	21.7%	
					Liver	15.5%	
					Lung	4.3%	
Luminal B	ER+ PR+ HER2−/+	10%	90.5%	44.7%	Bone	47.3%	More proliferative and generally worse prognosis than Luminal A Does not generally overexpress HER2; however, approximately 30% of patients do
					Brain	21.2%	
					Liver	25.7%	
					Lung	5.9%	
HER2 enriched	ER− PR− HER2+	4%	84.0%	37.9%	Bone	34.5%	Generally present intermediate-to-high grade tumours, associated with an aggressive course
					Brain	25.5%	
					Liver	31.7%	
					Lung	8.3%	
Basal like	ER− PR− HER2-	10%	76.9%	12.2%	Bone	36.4%	Also known as triple-negative BC Most aggressive subtype Large drop in survival rate once metastasis to distant sites has been diagnosed
					Brain	32.1%	
					Liver	22.4%	
					Lung	9.1%	

^a^Sourced from [46].

^b^Sourced from [45].

Metastasis formation is responsible for the majority of BC mortality [6]. BC’s ability to metastasise to distant sites early on in disease progression is a key determining factor for patients [7, 8]. Once engrafted, BC cells (BCCs) have the capacity to remain in a state of dormancy at these distant sites, either maintained as cancer stem cells (CSCs) or existing as disseminated tumour cells (DTCs), often remaining in this dormant state for decades before growing out into aggressive macrometastatic lesions [9, 10]. This phenomenon of dormancy allows the BCCs to evade primary treatment(s), eventually initiating a relapse diagnosis, complicating patient treatment. Unfortunately, 20–30% of BC patients may develop metastasis after diagnosis and primary tumour treatment, with approximately 90% of BC-related fatalities attributable to metastasis, thereby further fortifying BC as a clinical issue [11].

Dormancy does not have a clear biological definition and many have proposed classifying dormant phenotypes into cellular dormancy (when cells enter a reversible state of quiescence, remaining in the cell cycle stage G_0_) and tumour mass dormancy (the clustering of cancer cells, where cell death counterbalances proliferation) [12, 13]. This review will focus on cellular dormancy, although it is likely that these states co-exist in the same patients and that BCCs may fluctuate between them [14]. Dormancy is an adaptive, protective and opportunistic mechanism that malignant BCCs adopt to survive in their new microenvironment, allowing them to overcome the stresses that accompany this change [15]. The mechanisms or environmental cues that drive BCCs to enter and exit this state of cellular dormancy are ambiguous. For instance, studies examining BCC dormancy entry/ exit have included the analysis of a variety of classic cell signalling pathways, investigations into the effects of microenvironmental cues such as extracellular matrix stiffness and oxygen levels, the influence of stemness factors, as well as examinations into the effect of the immune system on BCC dormancy [9, 12, 14, 16–18].

Metabolic reprogramming is one of the classic hallmarks of cancer cells, with dysregulation of glucose metabolism, glutaminolysis and fatty acid (FA) synthesis providing the energy requirements for the rapid rate of cancer cell division [19–21]. Until recently, very little about the metabolic properties of lower energy demanding dormant BCCs was reported. This review will focus on recent progress made in our understanding of dormant BCC’s metabolic properties and the pathways involved in BCC entering, maintaining and exiting cellular dormancy, covering the influence of BC receptor status and potential therapeutic opportunities.

## BCC dormancy and carbon metabolism

The Warburg effect is a well-established phenomenon within the field of cancer research that was first defined by Otto Warburg in 1956. He described how, although cancer cells have a higher demand for energy than healthy cells, they can achieve high energy levels efficiently through the less effective metabolic process of aerobic glycolysis [22]. Normally, cells under a constant supply of oxygen undergo glycolysis to produce pyruvate from glucose, which is then oxidised into carbon dioxide via the process of oxidative phosphorylation (OXPHOS) in mitochondria. OXPHOS produces a highly efficient energy yield. However, when oxygen is absent, healthy cells will undergo incomplete oxidation of glucose, resulting in the production of lactate, entirely avoiding mitochondrial respiration and thereby producing a far lower energy yield [22]. Otto Warburg observed that, contrastingly, cancer cells can reprogramme their glucose metabolism, converting glucose into lactate via glycolysis, despite the presence of oxygen, even though this mechanism is a much less efficient source of energy [23]. A likely explanation for this cancer cell phenomenon, is the requirement of cancer cells for other metabolic end products that can accelerate cancer cell proliferation and progression. The role of the Warburg effect, also referred to as ‘aerobic glycolysis’, and OXPHOS in dormant BCCs is largely unexplored [24].

### Glycolysis shift during BCC dormancy/quiescence

Due to Warburg’s observations, the most notorious and thoroughly studied metabolic cancer cell alteration is an increased ‘aerobic glycolytic’ capacity. Nevertheless, glycolysis also has a role in preserving the ‘stemness’ of stem cells, ultimately maintaining stem cell pools and thereby also has a renowned role in maintaining less active cells [25, 26]. Therefore, we look at the recent studies that have focussed on the role of glycolysis on the dormant BCC phenotype, including the latest insights into the effect of glycolysis and lipid metabolism interplay on BCC activity.

**Fig. 1:**
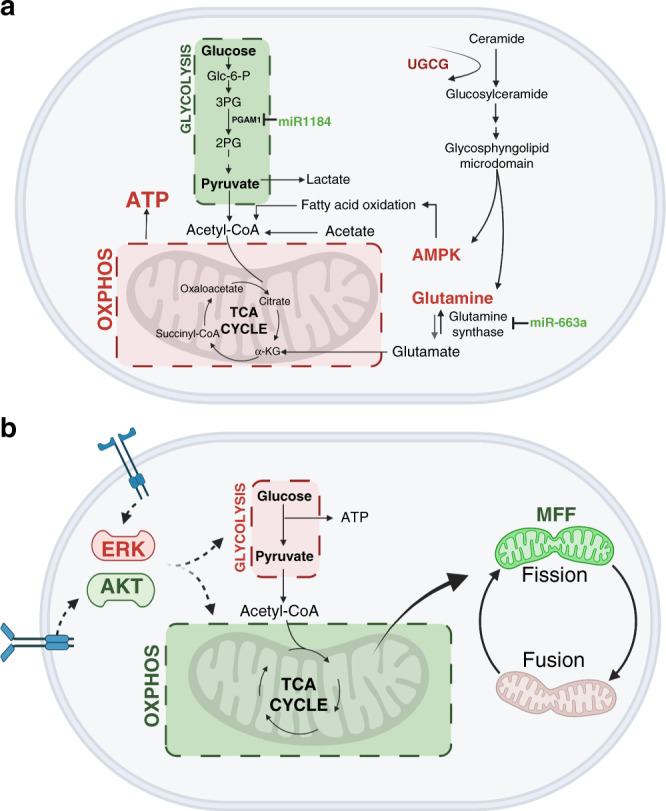
Schematic summary of glycolysis vs OXPHOS metabolic pathways associated with BCC dormancy. **a** Metabolic cues promoting a glycolytic shift for BCC dormancy. **b** Metabolic cues promoting an OXPHOS shift for BCC dormancy. Green colouring indicates an upregulation, red colouring indicates a downregulation.

Glucose metabolism is closely related to lipid metabolism, notably through the synthesis of FAs and glycerol [27]. An interesting recent study from Schömel et al. investigated the glycolytic and oxidative metabolic phenotypes of BCCs following the overexpression of the glycosphingolipid UDP-glucose ceramide glucosyltransferase (UGCG), a key enzyme of lipid metabolism (see Fig. 1a) [28]. UGCG activity results in the production of glucosylceramide, the precursor for all glycosphingolipids, and is related to poor prognosis in BC patients [29]. Schömel’s previous studies showed that the overexpression of UGCG leads to increased proliferation of BCCs, which led them to investigate what molecular mechanism was causing this proliferative advantage [29]. Their subsequent study demonstrated that via glutathione production, BCCs are able to fuel the tricarboxylic acid (TCA) cycle to sustain the proliferative capacity when UCGC was overexpressed (see Fig. 1a). This led the team to uncover that, when UGCG is overexpressed, MCF7s (ER+) experienced a BCC specific metabolic shift from a quiescent/aerobic state to an energetic state via an increase in both glycolysis and OXPHOS. Here they defined the MCF7s quiescent state via an observed lower energy demand. Additionally, the authors uncovered that UGCG overexpression altered cellular sphingolipid composition and that this contributed to observed increases in mitochondrial turnover and sustained levels of OXPHOS.

These studies highlight a close connection between BCC’s metabolic activity, glycosphingolipid metabolism and consequently their dormant/active state [28]. Although a strong focus on glycosphingolipid metabolism was presented, they also demonstrate a higher BCC dependence on glycolysis during dormancy and collectively attribute the BCC shift from dormant to proliferative via increases in OXPHOS. This work thereby highlights the potential for dormant BCCs employing a ‘reverse’ Warburg effect, using glycolysis to remain quiescent.

MicroRNAs (miRs) are small single-stranded non-coding RNAs that function in RNA silencing and post-translation gene expression regulation [30]. miRs have also been implicated in BC metabolic changes, through their regulation of key mRNAs involved in metabolism. A recent study by Muciño-Olmos et al. identified regulatory interactions between miRs and metabolic mRNAs representative for proliferative and quiescent BC phenotypes [31]. The miR and mRNA expression patterns of three-dimensional (3D) spheroid cultures of the BC cell line MCF7 (ER+) were compared. Here spheroids were grown over time, with earlier time points reflecting a proliferative BC model and later timepoints a quiescent BC model, as was demonstrated through spheroid proliferation and quiescence marker expression levels. Notably this study identified that quiescent stage MCF7 (ER+) spheroids have a lower expression of miRs that regulate glycosphingolipid and N-Glycan metabolism, suggesting that lipid metabolism is still active during dormancy. Furthermore, this study identified that quiescent stage spheroids had a decrease in expression of miR-3143, which regulates carbon metabolism via hexokinase-2, one of the first enzymes to regulate glycolysis, supporting the reliance of the dormant BCCs on glycolysis (see Fig. 1a). While these miR sets caused downregulation in the quiescent BCC models, the same sets were upregulated in their proliferative BC model, further suggesting a metabolic switch in the miRNA BC landscape which directly affects glycerophospholipid, N-Glycan and glucose metabolism [31].

Cancer cells need to rapidly increase their demand for amino acids for them to synthesise enough proteins, nucleotides and lipids, maintain redox homoeostasis and to fuel their energy requirements [32]. This is one of the main reasons cancer cells are thought to upregulate the glycolytic pathway during the Warburg effect [33]. As described, Muciño-Olmos et al. also observed that low expression of an array of miRs assisted in the rapid growth of their proliferative model. Specifically, aside from decreased miR-3143 expression, lower expression of miR-663a and miR-1184 respectively maintain a high expression of mRNAs that regulate glutamine synthetase (which catalyses the synthesis of glutamine from glutamate) and Phosphoglycerate Mutase 1 (PGAM1; which catalyses 3-phosphoglycerate to 2-phosphoglycerate during glycolysis). The low expression of these miRs thereby conserves the BCC amino acid levels (see Fig. 1a). Conversely, their dormant model showed that the overexpression of these miRs could downregulate amino acid synthesis, causing a dependence on glycolysis and lipid metabolism [31]. Thereby, this study suggests that lipid metabolism and glycolysis are associated with a shift in BCCs towards quiescence. These miR-mRNA interactions offer scope for developing BCC dormancy metabolism pathway learning, as well as novel targets for pharmacologically targeting BCC dormancy.

Contradictory to Warburg’s idea that cancer cells switch to glycolysis only to accelerate their proliferative capacity, these studies have demonstrated that BCCs can adapt their metabolic state to prefer glycolysis during a phase of dormancy.

### OXPHOS shift during BCC dormancy/quiescence

OXPHOS is recognised as being the most efficient energy yielding metabolic pathway, with ~18 fold higher efficiency of adenosine triphosphate (ATP) production relative to glycolysis [34]. Yet, 60% of cancer cells are believed to rely on aerobic glycolysis for their energy supply [32, 34]. While several studies have primarily focused on the impact of glycolysis on BCC dormancy, research has also highlighted the role OXPHOS plays during BCC dormancy.

In order to explore this area, consideration must be given to the microenvironments that host dormant BCCs. For instance, the bone marrow (BM) is the leading site for metastatic BC outgrowth, arising in approximately 60% of BC patients with metastatic disease [35], where up to 70% of BC patients harboured DTCs in their BM upon autopsy [36]. While the BM is a region rich in growth factors, its hostile hypoxic environment necessitates a high level of DTC metabolic plasticity. The organelle that deals with this plasticity is the mitochondrion. Impaired mitochondrial respiration was long believed to be the cause for increased cancer cell ‘aerobic respiration’; however, it is now established that (i) this is not the case for all cancers, and (ii) defects in mitochondrial respiration are not generally the cause of reinforced ‘aerobic glycolysis’ but that specific tumours that are mostly glycolytic can also retain high mitochondrial respiration levels [22, 37–39].

A study published by Buschhaus et al. recently demonstrated that quiescent BCCs in their 3D BCC-stromal BM model favoured OXPHOS metabolic activity [40]. Specifically, ER+ BC cell lines (MCF7 and T45D) were cultured with two BM mesenchymal stem cell lines (HS5 and HS27a), first in two-dimensional (2D) and then as 3D spheroids, generated using ultra-low attachment plates. Their co-culture included low BCC numbers to model the low number of DTCs in the BM, and the cell cycle status of BCCs in the 2D and 3D models was assessed with a fluorescent ubiquitination-based cell cycle indicator system or propidium iodide staining [40]. Their modelled BCC metabolic preference for OXPHOS metabolism could also be recapitulated in vivo. By injecting MCF7s (ER+) into the femoral artery of mice, facilitating preferential dissemination to the bone, ex vivo imaging was performed to determine the metabolic profile of the BCCs. The authors highlight that although the injected BCCs disseminated to the BM, at least some were proliferative instead of remaining dormant, highlighting the challenge in generating animal tumour dormancy models, particularly for ER+ BC. Nevertheless, using both the in vitro and in vivo models the authors could demonstrate that the BCCs in the BM exhibit an OXPHOS profile, which was accompanied by low extracellular signal-regulated kinase (ERK) activation and high Akt signalling: two of the most commonly activated signalling pathways in cancer, driving many cellular processes including proliferation, survival and drug resistance [41, 42]. Notably, low ERK activation is recognised as a general mechanism underlying cancer cell dormancy [16]. This study therefore demonstrates how there are bi-directional metabolic and signalling interactions between BCCs and stromal cells, thereby providing a platform for the dual targeting of metabolism and classic signalling pathways as a potentially effective approach for targeting dormant BCCs (see Fig. 1b) [40].

A combination of both glycolysis and OXPHOS were implicated for potential BCC dormancy in a recent study by Sánchez-alvarez et al. [43]. Here the authors showed that, by overexpressing the mitochondrial fission factor (MFF) in MCF7s (ER+), a quiescent BCC phenotype could be induced [43]. Using both 2D and 3D cultures, generated via ultra-low attachment plates, CSC marker levels and propagation were assessed to understand the level of quiescence activated [43]. Mitochondria are highly dynamic organelles, undergoing cycles of fission and fusion to maintain their morphology, distribution and size; these two dynamics are essential in regulating cell cycle, cell death and mitochondrial quality control [44]. MFF overexpression resulted in an abnormal activation or unbalanced mitochondrial fission that could interfere with both glycolysis and OXPHOS and stimulate quiescent programs, thus demonstrating that both pathways may be required for dormancy (see Fig. 1b) [43]. Suppression of mitochondrial fission, and possibly an exploitation of this, may represent a useful tool in the activation of BCC quiescence programs.

Current studies do not seem to find a common consensus on whether glycolysis or OXPHOS is the main driver of the dormant BCC phenomenon. In general, these studies have focused on the use of MCF7s (ER+) as a BC model. However, as BC is such a multifaceted and heterogenous disease, an exploration of pathways feeding these two key metabolic processes within different BC subtypes may provide a clearer metabolic picture of explicit BCC subset dormancy programs.

## Other ER+ dormant BC metabolic pathways

The reactivation of dormant BCCs at metastatic sites is hypothesised to be a mechanism of both early and late BC recurrence, where the elapsed time after cancer recurrence upon successful treatment is dependent on the BC subtype [5, 7, 45]. For instance, triple-negative BC patients typically experience recurrence after 1–2 years, while Luminal BC (which presents with ER+ receptor status) is associated with much later recurrence ranging between 5 and 20 years after remission, suggesting that ER+ BCCs undergo a lengthy period of dormancy [45, 46]. This is supported by the fact that ER+ BC recurrence occurs almost entirely at the site of metastasis [47].

Luminal BC (ER+) patients are typically treated with surgical resection proceeded by 5≤ years of adjuvant endocrine therapy, targeting ER activity either by inhibiting the ER directly (e.g. via tamoxifen) or by suppressing ER biosynthesis (e.g. via aromatase inhibitors). However, these therapeutic approaches unfortunately do not address residual dormant BCCs [46, 48–50].

### The role of AMPK and fat metabolism in ER+ BC cell dormancy

AMPK (5’ adenosine monophosphate-activated protein kinase) is a cellular energy sensor that can promote catabolism in response to stress signals and a high AMP:ATP ratio (reflecting a compromised energy status). Therefore, it is involved in a plethora of cellular activities, such as cell growth and metabolism [51].

**Fig. 2:**
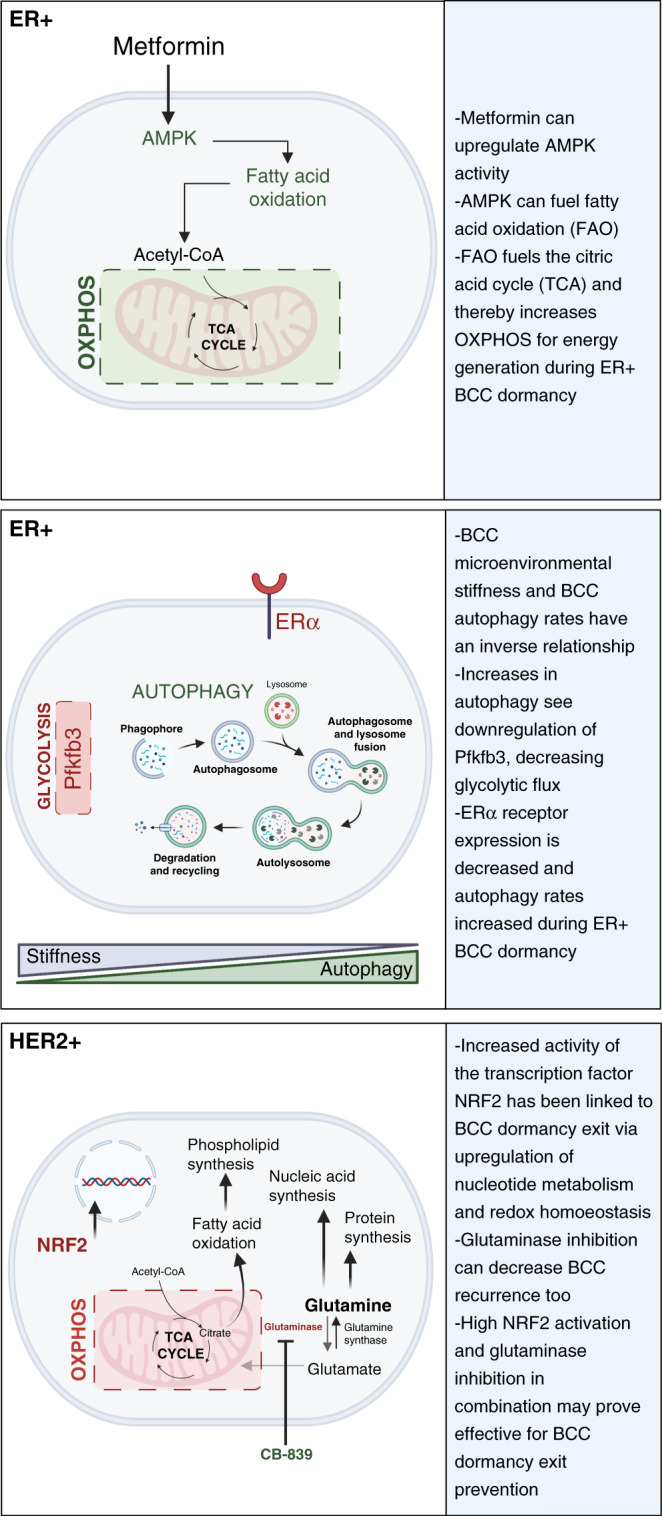
An overview of receptor-specific metabolic influences on BCC dormancy. These panels represent different BCC metabolic activities reported in either ER+ or HER2+ BC dormancy models. Green colour is indicative of an upregulation and red colouring is indicative of a downregulation [56, 68, 73, 77].

Recently, AMPK has been uncovered as a mediator of ER+ BCC dormancy (see Fig. 2). A set of studies aimed to investigate why dormant ER+ BCCs survive despite being starved of oestrogen during chemotherapy. When the anti-diabetes drug metformin was being tested as an anti-cancer treatment, it was found to activate AMPK. Metformin also slowed the oestrogen-driven growth of BCCs, though it promoted the persistence of oestrogen-deprived cells via increased fatty acid oxidation (FAO), which in turn fuelled OXPHOS. This therefore suggests that during oestrogen starvation, if AMPK is activated, fat metabolism gets upregulated, which can in turn produce the energy needed for the dormant ER+ cells to survive [52–56].

Importantly, Hampsch et al.’s preclinical studies have shown that oestrogen withdrawal in their in vivo mouse model causes an activation of AMPK and upregulates FAO [56]. Specifically, BCCs have been shown to survive extended oestrogen withdrawal in a non-palpable, dormant state while retaining tumorigenicity. Oestrogen withdrawal was thereby performed on mice to mimic ER-suppressing aromatase inhibitor therapy. Using non-invasive bioluminescence imaging, they showed that the 60–90-day window was optimal for studying stable residual tumour cells. Further to this, they also assessed proliferation marker expression of these tumour cells to additionally describe their dormant model. Unsurprisingly, a high dietary fat intake promoted the survival of ER+ BCCs in these mice. Further to this, by both inhibiting BCC’s FAO with anti-anginal drugs (which block fat metabolism) and via pharmacological or genetic inhibition of AMPK, the researchers were able to induce the clearance of dormant BCCs, highlighting the dependency of dormant ER+ BCCs on FAO [56].

In summary, the preclinical model of dormant vs active ER+ tumours used in these particular studies demonstrate AMPK activation as a key driver of persistent ER+ BCC dormancy. BCC persistence was further enhanced with the treatment of metformin, while this increase in persistence could be reduced by AMPK inhibition [56]. As the inhibition of AMPK could also promote tumour growth, this therefore does not present a viable option for targeting dormant ER+ BCC. A more practical therapeutic strategy could be to repurpose FAO inhibitors, which have already been clinically approved for angina treatment [51, 56]. These studies have also provided evidence that high dietary fat intake increased the survival of dormant BCCs, indicating that diet can have a clear impact on patient outcomes. Interestingly, a reduction in ATP levels and subsequent AMPK activation not only upregulates FAO but also upregulates the important metabolic pathway of autophagy (reviewed in [57]).

### Autophagy, tissue mechanics and the ERα receptor

Autophagy defines a highly conserved and tightly regulated cellular recycling mechanism. In essence, double membrane vesicles called autophagosomes form to gather misfolded and/or mutated proteins, damaged or aged organelles and subsequently fuse to the lysosome leading to the degradation of gathered components [58, 59]. This mechanism therefore has a key regulatory role in metabolism, maintaining the energy balance during times of limited nutrient availability via the degradation of energy stores such as proteins, lipid droplets and glycogen [58]. Autophagy is upregulated in cancer cells in order to help meet their high demand for nutrients; nevertheless, the role of autophagy in cancer progression is somewhat paradoxical. Autophagy has been found to promote cancer progression and cell growth though the provision of nutrients; however, it can also suppresses tumour growth and development through the reduction of oxidative stress and the removal and degradation of dysfunctional macromolecules [58, 60, 61]. Importantly, autophagy has been reported to increase the survival of dormant cancer cells (see Fig. 2).

A study in 2018 demonstrated how both pharmacologic and genetic inhibition of autophagy impaired the survival of dormant BCCs in vitro and in vivo. Here their in vitro model consisted of both murine and human cell lines, cultured in 3D using basement membrane extract matrices, with dormancy exhibited through cell cycle stage analysis and the expression levels of p16 and p27, both expressed during dormancy. Their in vivo model consisted of D2A1 and D2.0R tumour cell lines being injected into the mammary fat pad of mice, which were shown to disseminate to the lungs, where they remained dormant [62–64]. Interestingly, these models demonstrated that the inhibition of autophagy had a minimal effect on metastatic outgrowth in the BCC models employed, once the BCCs had transitioned into a more proliferative state. The clearance of damaged mitochondria and the maintenance of redox homoeostasis in the early stages of the respective model’s BCC metastatic colonisation was shown to play an important role [64].

Cancer cell behaviour is influenced by the cellular microenvironment. Tissues where relapse is commonly detected, including the BM, brain and lung, tend to be softer tissues than the original mammary gland. The link between the stiffness of secondary metastatic sites and disseminated BCC metabolic properties is thought to play a key role in regulating BCC dormancy and entry/exit [65].

To investigate the influence of microenvironmental stiffness on active/ dormant BCCs and their energy sources, Ovadia et al. developed a bioinspired 3D synthetic matrix model. Here they found that BCC dormancy and outgrowth depended on the 3D model’s matrix composition and BCC subtype, where dormancy was defined via a combination of cell viability, metabolic activity (Alamar blue) and through cell proliferation (EdU incorporation) assays [66]. ER+ BCC cells demonstrated dormancy over continued growth in their different matrix densities, whereas triple-negative BCCs were not observed to undergo any long-term dormancy in the same models [25]. This reflects the long-term dormancy experienced in ER+ Luminal A/B BC vs the early relapse associated with triple-negative BC [4, 67]. Interestingly, ER+ BCC cultured in the long-term 3D bioinspired matrices model also showed an upregulation in autophagy as a source of energy. This study thereby highlights a well-defined platform for investigating the metabolic properties of receptor-specific late BCC recurrence, including the effect of matrix density on autophagy and dormancy [66].

A further study examined the influence of matrix stiffness on BCC metabolic dependence and dormancy. It showed that BCCs seeded into a soft microenvironment were more resistant to tamoxifen, the ER inhibitor, as a result of increased autophagy rates and a decrease in the expression of oestrogen receptor α (ERα) [68]. Conversely, decreases in autophagy were observed in BCCs cultured on stiff substrata. The genetic downregulation or pharmacological inhibition of autophagy in ER+ BCC cultured within soft substrata increased their response to tamoxifen. The therapeutic outcomes of this research further highlights the importance of tissue mechanics in ER+ BCCs ability for long-term survival by sourcing energy via autophagy [68].

Control of glycolytic rate plays a central role in cellular homoeostasis, with a dysregulation being a hallmark of cancer (as discussed in previous section ‘BCC dormancy and carbon metabolism’) [24, 69]. 6-Phospho-fructo-2-kinase/fructose 2,6-biphosphatase 3 (Pfkfb3) is not only a protein critical in regulating the rate of glycolysis, acting to sustain a high glycolytic rate, but also has a role in promoting cell cycle progression, through the G_1_ and S phases and by suppressing apoptosis [70–72]. Pfkfb3 is upregulated in numerous human cancers and was found, when upregulated in BCCs, to predict for reduced relapse-free survival [73, 74].

Pfkfb3 expression and autophagy exhibit an inverse relationship [75]. In a study from 2019, dormant BCCs presented with low Pfkfb3 expression and high autophagy rates vs the opposite phenotype for metastatic/proliferative BCCs [73]. Specifically, both genetic and pharmacological inactivation of autophagy caused an upregulation of Pfkfb3 in dormant BCCs that resulted in BCC proliferation and outgrowth, while conversely depleting Pfkfb3 expression in BCCs reduced survival and outgrowth. Here the authors used an established model of BC dormancy that comprises dormant D2.OR cells and metastatic D2.A1 cells originally derived from BALB/c mice. Thereby, they achieved both an in vitro 3D culture dormancy model and in vivo dormant BC model of lung metastasis. The authors suggest scope for stratified therapies depending on patient Pfkfb3 expression and autophagy status. Furthermore, they highlight that the pharmacological activation of autophagy could counteract Pfkfb3 expression, preventing relapse by maintaining continuous BCC dormancy [73].

Intriguingly, Pfkf3b is regulated by AMPK to mediate glycolysis, even though AMPK is also able to stimulate autophagy (see Fig. 2) [75]. Therefore, understanding the links between AMPK, autophagy and Pfkfb3 during BCC dormancy provides scope for research. Therapeutic approaches could potentially target AMPK and autophagy as part of primary BC treatment or as an adjuvant therapy, which may help to reduce the viable dormant ER+ BCC population.

## Her2 dormant BC metabolism

*HER2* can act as an oncogene and is therefore a marker of poor BC prognosis, being overexpressed and/or amplified in approximately 15–30% of BC patients [76]. Her2-positive BC tumours progress faster and more aggressively than luminal A/B BC subtypes (ER+). Her2 has also been shown to affect several metabolic pathways. For instance, inhibition of Her2 causes an instant fall in glucose uptake [77]. Further to this, cells that can survive the loss of oncogenic Her2 upregulate FAO pathways, thereby directly increasing reactive oxygen species (ROS) levels [78]. Potential Her2 metabolic therapeutic targets are reviewed in [79].

### NRF2 pathway and recurrence

To gain an insight into how Her2 oncogenic pathways can promote metabolic adaptations during BC dormancy and reactivation, Fox et al. used a transgenic mouse model of Her2-driven BC [77]. This dormancy model was validated in 3D non-adherent conditions, where primary Her2-driven tumour cells were shown to be dependent on Her2 for growth and survival, yet a population of cells could survive Her2 downregulation, persisting in a viable, non-proliferative state [77].

Her2 inhibition led to ROS dependent cell death, which suggested that dormant tumour cells that survive Her2 downregulation treatments may have intrinsic antioxidant pathways activated. This led the authors to recognise that the NRF2 (nuclear factor (erythroid-derived 2)-like 2 or (Nfe2l2)) pathway was a key moderator of metabolic reprogramming, as a direct consequence of oncogene inhibition. NRF2 is a transcription factor that regulates the cellular adaptive antioxidant response and has been shown to be a driver of tumour progression, therapy resistance and metastasis [77]. This key study found that NRF2 promoted the recurrence of dormant BCCs by encouraging nucleotide metabolism and redox homoeostasis; nucleotide metabolism is defined as the process during which DNA and RNA are synthesised and degraded and redox homoeostasis is the endogenous cellular response to challenges that generate electrophiles, both processes crucial for maintaining cellular health and energy levels [30, 77].

An increase in glutamine metabolism (glutaminolysis) is typical for cancer cells, enabling and maintaining their high energy demand by replenishing the TCA cycle and providing nitrogen, sulphur and carbon for proliferating cells [30]. Glutamine is processed by glutaminase, a mitochondrial enzyme active in tumours, producing glutamate and α-ketoglutarate, which then progress to produce carbon and nitrogen for the biosynthesis of proteins, FA and nucleic acids [80]. Moreover, limiting glutaminase activity results in a decreased growth rate in tumour cells [81]. Notably, Her2+ BCCs are highly glutamine dependent [77, 79]. Fox et al. subsequently also uncovered that glutaminase inhibition could prevent the reactivation of dormant Her2 BCCs. Specifically, the CB-839 glutaminase inhibitor resulted in a fourfold decrease in BCC recurrence (see Fig. 2) [77]. Recurrent tumours with high NRF2 activation were sensitive to glutaminase inhibition, therefore presenting a potentially novel therapeutic strategy for treating recurrent tumours by controlling relapse after therapy.

This is not the first study to link Nrf2 expression with BCC metabolism. In 2019, Zhang et al. found that the overexpression of Nrf2 in MCF‐7 (ER+) and MDA‐MB‐231 (ER−) cells promoted the expression of glucose‐6‐phosphate dehydrogenase (G6PD), (which is involved in the pentose phosphate pathway, running parallel to glycolysis to generate pentose, NADH and nucleotide precursors) and hypoxia-inducing factor α (HIF1α; which is involved with activating glycolysis during cellular hypoxia) and that this consequently caused an upregulation of the Notch signalling pathway. This accordingly affected BCC migration via the epithelial–mesenchymal transition pathway [82]. This study shows how the interaction of Nrf2 expression with glucose metabolism can affect the metastatic and proliferative activity of BC cell lines negative for Her2 too.

Although recent research into Nrf2 has advanced our understanding of Her2 BCC dormancy metabolism, there are limited studies in this area. Constitutive Her2 expression has been shown to increase Pfkfb3 expression and thereby upregulate glycolysis. It would therefore be interesting to evaluate the effects of Pfkfb3 and its effect on autophagy in Her2 BCC dormancy.

## Concluding remarks

Here we conclude that a variety of different pathways have been linked to BCC dormancy entry/exit. Due to the heterogeneity of BC, we see that there is potential for different metabolic mechanisms and pathways influencing BCC activity and that this may be a result of hormone receptor status and BC subtype. Notably, there appears to be no research investigating the link between progesterone status and dormant BCC metabolism. Furthermore, there have been limited comments on the metabolic properties of dormant basal-like BCCs (which present as ER, PR and Her2 negative), which remains to be fully explored. Basal-like BCCs have the fastest recurrence rate and are associated with early relapse, thereby exemplifying how BCCs’ ability to transition between dormant/active states is of key importance, rather than their duration of dormancy. Fundamentally, as receptor status seems to underly this, a comparison of metabolism during basal-like and luminal BC dormancy entry/exit will prove insightful. Such a study would help clarify whether there are shared metabolic features that govern the transition in and out of BC dormancy, which could potentially be used to design a comprehensive BC dormancy treatment.

Currently, the only clinical trial targeting both dormant and active BCCs is a proof-of-concept trial. This ER+ BC trial uniquely targets the upregulation of the cyclin-dependent kinase 4/5 pathway and immune evasion, which have been shown to influence BCC dormancy exit, in addition to autophagy, for which a dormancy metabolic mechanism has been described (Clinicaltrials.gov identifier: NCT04841148) [83]. The outcome will ultimately assess whether a reduction in recurrence can be achieved through dual targeting of dormant BCC metabolic pathways, as well as active BCC pathways. The result of this trial is pending yet will hopefully highlight scope for further dual targeting trials.

A recent review provides some thoughtful comments, suggesting evidence for the avoidance of BCC dormancy/outgrowth achieved through mitochondrial ATP-depletion therapy [84]. Focussing therapies at inhibiting the mitochondrial ATP synthase could therefore also be an important BCC dormancy metabolic consideration.

Finally, this review highlights the need to classify the definition of an in vitro and in vivo dormant BCC model, with the variety of models and the divergencies in dormancy evidence presented here illustrating this shortfall. Cellular dormancy is believed to be the result of cancer cell growth arrest. Thereby, studies should standardise examining expression levels of the proliferation marker Ki67 (which is not expressed during G0), as this is also a prognostic BC marker [85]. Additionally, providing transcriptional profiles for each dormancy model could elucidate dormant/recurrent signatures across BC subtypes, delivering significant prognostic value. This will ultimately lead to an improved understanding of the relationship between BCC dormancy entry/exit and BCC metabolism, paving the way for better relapse-free BC therapies.

## Data Availability

Not applicable.
